# The clinical and integrated management of COPD. An official document of AIMAR (Interdisciplinary Association for Research in Lung Disease), AIPO (Italian Association of Hospital Pulmonologists), SIMER (Italian Society of Respiratory Medicine), SIMG (Italian Society of General Medicine)

**DOI:** 10.1186/2049-6958-9-25

**Published:** 2014-05-19

**Authors:** Germano Bettoncelli, Francesco Blasi, Vito Brusasco, Stefano Centanni, Antonio Corrado, Fernando De Benedetto, Fausto De Michele, Giuseppe U Di Maria, Claudio F Donner, Franco Falcone, Carlo Mereu, Stefano Nardini, Franco Pasqua, Mario Polverino, Andrea Rossi, Claudio M Sanguinetti

**Affiliations:** 1 General Practitioner, Brescia, Italy; 2 Respiratory Diseases, Cà GrandaOspedale Maggiore Milano Foundation, ‘UniversitàdegliStudi’ of Milan, Milan, Italy; 3 Respiratory Diseases, ‘UniversitàdegliStudi’ of Genua, Genua, Italy; 4 Respiratory Diseases, San Paolo Hospital, ‘UniversitàdegliStudi’ of Milan, Milan, Italy; 5 Intensive Therapy and Thoracic Pathophysiology, Careggi Hospital, Florence, Italy; 6 Pneumology Unit, SS. Annunziata Hospital, Chieti, Italy; 7 Pneumology I and Respiratory Pathophysiology Unit, A. Cardarelli Hospital, Naples, Italy; 8 School of Specialization in Respiratory Diseases, Pulmonology Unit and Sleep Medicine, Department of Clinical and Molecolar Biomedicine, University of Catania, Catania, Italy; 9 Mondo Medico, Multidisciplinary and Rehabilitation Outpatient Clinic, Borgomanero, NO, Italy; 10 Department of Pneumology, GVM Care & Research, Villalba & Villa Torri Hospital, Bologna, Italy; 11 Pneumology Department and Medical Field Department, ASL 2, Savona, Italy; 12 Pulmonary and TB Unit, Vittorio Veneto General Hospital, - ULS 7- Veneto Region, Vittorio Veneto, TV, Italy; 13 Pneumology Rehabilitation, IRCCS S. Raffaele, Rome, Italy; 14 North Salerno Lung Diseases Pole, ASL SA, Salerno, Italy; 15 Pneumology Unit, University and General Hospital of Verona, Verona, Italy; 16 Past Director, Pneumology Unit-UTIR, San Filippo Neri General Hospital, Rome, Italy

**Keywords:** COPD, Integrated care, Management

## Abstract

COPD is a chronic pathological condition of the respiratory system characterized by persistent and partially reversible airflow obstruction, to which variably contribute remodeling of bronchi (chronic bronchitis), bronchioles (small airway disease) and lung parenchyma (pulmonary emphysema). COPD can cause important systemic effects and be associated with complications and comorbidities. The diagnosis of COPD is based on the presence of respiratory symptoms and/or a history of exposure to risk factors, and the demonstration of airflow obstruction by spirometry. GARD of WHO has defined COPD "a preventable and treatable disease". The integration among general practitioner, chest physician as well as other specialists, whenever required, assures the best management of the COPD person, when specific targets to be achieved are well defined in a diagnostic and therapeutic route, previously designed and shared with appropriateness. The first-line pharmacologic treatment of COPD is represented by inhaled long-acting bronchodilators. In symptomatic patients, with pre-bronchodilator FEV1 < 60% predicted and ≥ 2 exacerbations/year, ICS may be added to LABA. The use of fixed-dose, single-inhaler combination may improve the adherence to treatment. Long term oxygen therapy (LTOT) is indicated in stable patients, at rest while receiving the best possible treatment, and exhibiting a PaO_2_ ≤ 55 mmHg (SO_2_ < 88%) or PaO_2_ values between 56 and 59 mmHg (SO_2_ < 89%) associated with pulmonary arterial hypertension, *cor pulmonale*, or edema of the lower limbs or hematocrit > 55%. Respiratory rehabilitation is addressed to patients with chronic respiratory disease in all stages of severity who report symptoms and limitation of their daily activity. It must be integrated in an individual patient tailored treatment as it improves dyspnea, exercise performance, and quality of life. Acute exacerbation of COPD is a sudden worsening of usual symptoms in a person with COPD, over and beyond normal daily variability that requires treatment modification. The pharmacologic therapy can be applied at home and includes the administration of drugs used during the stable phase by increasing the dose or modifying the route, and adding, whenever required, drugs as antibiotics or systemic corticosteroids. In case of patients who because of COPD severity and/or of exacerbations do not respond promptly to treatment at home hospital admission should be considered. Patients with "severe" or "very severe" COPD who experience exacerbations should be carried out in respiratory unit, based on the severity of acute respiratory failure. An integrated system is required in the community in order to ensure adequate treatments also outside acute care hospital settings and rehabilitation centers. This article is being simultaneously published in *Sarcoidosis Vasc Diffuse Lung Dis* 2014, 31(Suppl. 1);3-21.

## Introduction

Respiratory diseases currently represent the second cause of death worldwide, though they are underestimated. Because of the increasing life span of general population and the persisting smoking habit, chronic obstructive pulmonary disease (COPD) is expected, based on the current trends of incidence, to become the third cause of death worldwide by 2020.

**Figure 1:**
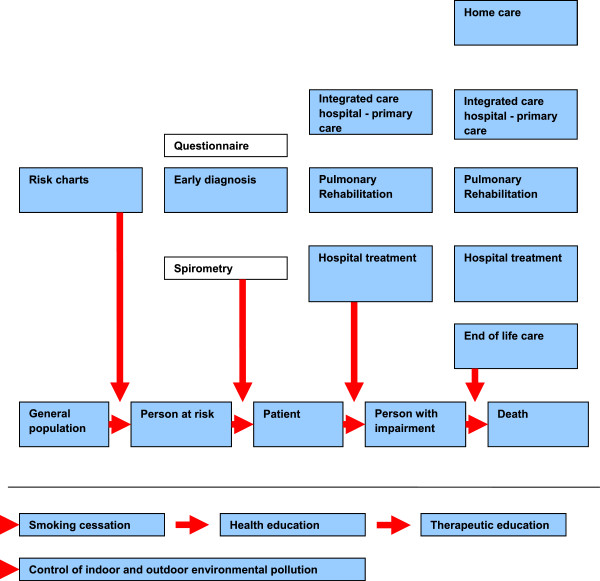
General guidelines for prevention and care of chronic respiratory diseases.

Symptoms of COPD, cough, phlegm, and dyspnea, are often overestimated and the diagnosis is made only in the sixth decade of life, when the patients are already in the moderate-to-severe stage and lung function is impaired. Frequently, the diagnosis is made when the patient is hospitalized because of an exacerbation, which points to the inadequacy of the current standards for diagnosis and treatment.This document is an update of the COPD guidelines published in Italy by the National Agency for Regional Health Services (AGE.NA.S.) and is intended to offer an instrument for practical and integrated management of COPD, aiming at appropriateness of diagnosis and therapy. The document is addressed to pulmonologists and other specialists working either inside or outside hospitals, general practitioners, other health professionals, patient’s associations, and institutions at national, regional, or local level. Figure 1 shows general guidelines for COPD management.

The document has been prepared by a working group appointed by the three major national respiratory societies (AIMAR, AIPO e SIMeR) and the Italian Society of General Medicine (SIMG). Representatives of the Italian Ministry of Health and AGE.NA.S. were involved as external independent observers to warrant for ethical, social and solidarity principles.

The reference list of each chapter is meant to be essential and not exhaustive regarding the information given.

### Methodological note

Health Information is the spread of any health-related information, without assessment of the impact that the message has on addressees. It can be done by direct verbal messages, movies, brochures, posters, or other media (e.g. web) [1,2].

Health Education is a set of general information on behavioral norms, knowledge, attitudes, habits and values that contribute to expose to or protect from harm to health. It applies to both healthy and sick people. It includes general norms that can be learnt in different contexts, such as family, school, society and health organizations [1,2].

Therapeutic Education is a set of educational activities in favor of specific categories. It is put into action by transmission of knowledge, training to achieve skills and promote behavioral changes. It requires that educators have specific knowledge of science and communication, with proficiency in the use of specific methodologies and verification of results [3].

The goal of Health Education is to improve the efficacy of treatments for chronic pathological conditions through the active and responsible participation of patient to therapeutic plan. The improvement of life-style in support of treatments and the participation in the choice of changes account for a greater efficacy of treatments and psycho-physical personal well-being.

## COPD definition and diagnosis

### Definition

COPD is a chronic pathological condition of the respiratory system characterized by persistent and not fully reversible airflow obstruction, to which variably contribute pathologic change of bronchi (chronic bronchitis), bronchioles (small airway disease) and lung parenchyma (pulmonary emphysema). COPD is caused by the inhalation of noxious agents, mainly tobacco smoke, which cause chronic inflammation by various mechanisms. Clinical manifestations are chronic cough and phlegm, dyspnea and reduced exercise tolerance.

### Pathophysiology

Chronic airflow obstruction is the results of a combination of various abnormalities differing for type, site, severity and extent. In a number of patients, perhaps the majority, the reduced caliber of airways, mainly the more peripheral ones with a diameter < 2 mm [1,4], due to inflammation, mucous hypersecretion and remodeling, and the destruction of lung parenchyma may cause:

• *Static lung hyperinflation,* i.e., an increase in the volume at which lung and chest wall are at static equilibrium, due to reduction of lung elastic recoil pressure.

• *Dynamic lung hyperinflation,* i.e., an increase of end-expiratory lung volume above the static equilibrium volume, due to increased airflow resistance. In more severe patients it may be present even at rest; in less severe ones it occurs when either minute ventilation is increased, e.g., during exercise, or airflow resistance is further increased, e.g., during exacerbations.

• *Ventilation-Perfusion mismatching*.

COPD can cause important systemic effects and be associated with complications and comorbidities, common in elderlies or more severe cases. COPD is the commonest cause of chronic respiratory failure and disability.

### Diagnosis

The diagnosis of COPD is based on a history of exposure to risk factors, either associated or non-associated with respiratory symptoms, and the demonstration of airflow obstruction by (simple) spirometry and additional pulmonary function tests.

**Figure 2:**
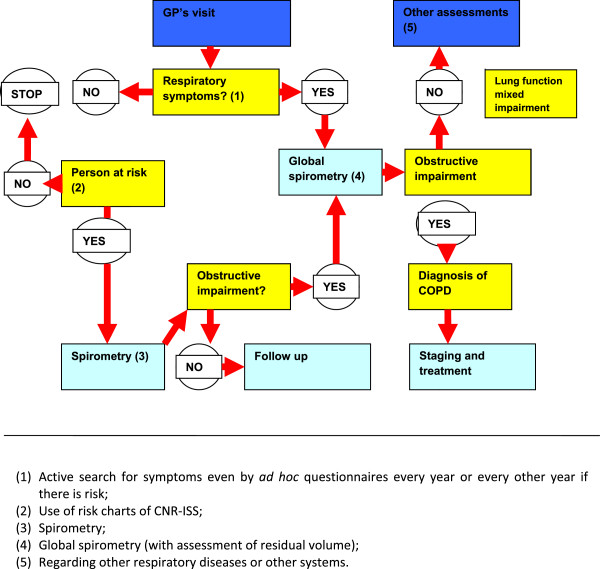
Proposal of diagnostic procedure and case finding for COPD.

A ratio of 1-s forced expiratory volume (FEV_1_) to vital capacity (FEV_1_/VC) remaining below the limit of normality 15–30 min after the inhalation of a bronchodilator (salbutamol 400 μg) is sufficient to confirm the diagnosis. The fixed ratio FEV_1_/FVC < 70%, frequently used as a lower limit of normality yields falsely negative results in subjects aged < 50 years and falsely positive results in those aged > 50 years [5-7]. Therefore, the use of the 95° percentile of predicted FEV_1_/VC for age and sex is recommended. It must also be noted that the vital capacity measured with a forced expiratory maneuver (FVC) may be underestimated compared with that measured with a slow maneuver (VC). The functional abnormality of COPD can be characterized by comprehensive physiological studies, which should be included in the diagnostic process in addition to simple spirometry. These include the measurement of absolute lung volumes, particularly residual volume [8] and functional residual capacity, and lung diffusion capacity for carbon monoxide (DL_CO_) to evaluate the degree of lung hyperinflation, gas trapping, and the presence of pulmonary emphysema [9,10].Spirometry is a necessary investigation to confirm the diagnosis of COPD and represents, together with symptoms, quality of life, frequency and severity of exacerbations, and frequency of hospitalizations, a major criterion to evaluate clinical condition and to make choice of the most appropriate treatment. If the subject is unable to perform acceptable spirometry maneuvers, the doctor should treat him/her as "suspected COPD" based on history and clinical data. Persisting or recurrent episodes of cough, sputum for several consecutive days and respiratory infections (cold, flu-like syndrome, bronchitis) with slow resolution and, mainly, dyspnea disproportioned to effort or age are signs that must be reported to the general practitioner. This is in charge of recording the respiratory symptoms of his/her patient (also using the respiratory risk chart for COPD) and referring him/her for appropriate diagnostic investigations, particularly spirometry and/or pulmonologist’s visit. General practitioners are also in charge for active search of new cases, through the use of questionnaires suitable for case finding among individuals potentially affected by COPD. The use of an electronic record carefully updated with patient’s data enables the general practitioner and the specialist to monitor disease progression. Scientific societies must be active in pursuing this goal, while the central and local Institutions must sensitize the general population. Figure 2 shows the diagnostic procedure for COPD.

## Integrated hospital and primary care of the patient with stable COPD

The Global Alliance against Chronic Respiratory Diseases (GARD) of the World Health Organization (WHO) has defined COPD "a preventable and treatable disease"; hence a great responsibility is cast on government and health local Authorities, on Hospital Chest Physicians, on primary care Physicians and staff and, last but not least, on scientific societies.

GARD recommends that National Health Systems works to get the following goals:

• total tobacco control (as a cause of COPD) and control of other (less relevant) risk factors;

• health education driven actions toward general population for primary and secondary prevention;

• COPD screening with simple and affordable means;

• professional education of health staff to risk factors (primary prevention), to screening procedures (also identifying individuals with personal characters putting them at risk of developing COPD) and to optimal and sustainable treatments;

• patient education to self management of COPD;

• a COPD care network of health staff aimed at integrating the current fragmented ultra-specialistic knowledges using well known, shared guidelines and protocols.

### Follow up of stable COPD patient

#### Key points

The management of a person suffering from COPD can reach high complexity levels during the advanced stages when reduced gas exchanges, reduced exercise capacity, increasing breathleness and important cardio-vascular, metabolic, oncologic and psychiatric comorbidities can be associated with a severe functional deficit.

This sub-population of patients with advanced COPD, even if a small proportion of the total population, is responsible for the highest use of heath system resources, having a strong impact on NHS. It requires a complex management, coordinated between specialized and primary care. The best management can be attained with a careful integration among chest physician, general practitioner as well as other specialists, whenever required.

For each health professional specific tasks should be defined, which should be included in diagnostic and therapeutic pathways, previously designed and shared for each severity stage of the disease.

Table 1 shows the proposals for the follow up of COPD, according to different severity stages. Which type of control, by which health professional and at what time is carried out are also specified.

**Table 1:** Planning COPD monitoring

**Planned actions**	**Monitoring chronic bronchitis (without flow limitation) or mild COPD (FEV**_**1**_**/CV < LLN and FEV**_**1**_ **> 80%) without symptoms**	**Monitoring COPD with FEV**_**1**_ **< 80% and/or exercise dyspnea and/or comorbidities**	**Monitoring COPD with FEV**_**1**_ **< 60% and/or exercise dyspnea and/or frequent exacerbations and/or comorbidities**	**Monitoring COPD with FEV**_**1**_ < 50% with respiratory insufficiency and comorbidity**
**Timing**	**Every other year**	**Every year**	**Every year**	**Every year**
Smoking cessation, if a smoker	Every physician/nurse or smoking cessation clinic	Every physician/nurse or smoking cessation clinic	Every physician/nurse or smoking cessation clinic	Every physician/nurse or smoking cessation clinic
Clinical check (including Body Mass Index, questionnaires and assessment of risk factors)	Chest physicians and general practitioner	Chest physicians and general practitioner	Chest physicians and general practitioner	Chest physicians and general practitioner
Pulsoximetry	Chest physicians and general practitioner	Chest physicians and general practitioner	Chest physicians and general practitioner	Chest physicians and general practitioner
Flow-volume curve	Chest physicians and general practitioner	Chest physicians and general practitioner	Chest physicians and general practitioner	Chest physicians and general practitioner
Chest physician consultation	Chest physician	Chest physician	Chest physician	Chest physician
Full spirometry	Respiratory function unit*	Respiratory function unit	Respiratory function unit	Respiratory function unit
Diffusion test (DLCO)		Respiratory function unit*	Respiratory function unit*	Respiratory function unit
Chest-X-ray*	Radiology	Radiology	Radiology	Radiology
EKG*	Chest physicians and general practitioner	Chest physicians and general practitioner	Chest physicians and general practitioner	Chest physicians and general practitioner
EKG cardiac ultrasound		Specialized unit	Specialized unit	Specialized unit
Blood gas analysis (BGA)			Respiratory function unit	Respiratory function unit
6-min walking test		Respiratory function unit	Respiratory function unit	Respiratory function unit
Nocturnal pulsoximetry*		Respiratory function unit	Respiratory function unit	Respiratory function unit
Other consultation and/or tests*		Other consultation and/ or tests*	Other consultation and/ or tests*	Other consultation and/ or tests*
		General practitioners are in charge of chronic treatment monitoring: Every six months he/she checks the clinical situation in own clinic. He/she carries out a pulsoximetry at each exacerbation and the following 2 months. Refers the patient to a consultation in case of persistent worsening	General practitioners are in charge of chronic treatment monitoring: Every three months he/she checks the clinical situation in own clinic. Refers the patient to a consultation in case of exacerbation. Chest physician has in charge the patient until the recovery of the steady state	General practitioners are in charge of chronic treatment monitoring. Every two months he/she checks the clinical situation in own clinic. Quickly refers the patient to a consultation in case of exacerbation or complaint of new symptoms/signs. Chest physician has in charge the patient until the recovery of the steady state and monitors the comorbidities, using the proper referrals

(*) when needed (**) patient in OLTT deserves BGA and clinical check at least every six-month.

### Management of COPD in stable state

#### Key points

COPD is a chronic and complex condition which usually tends to worsen over time. To control this evolution it is necessary to quit smoking and eliminate risk factors as well as to comply with the proper treatment (both pharmacologic and non-pharmacologic) which should be continued over time and tailored to the individual person needs, using clinical sings and functional test to step up the treatment.

Also comorbidities (mainly cardiovascular and metabolic) and complications should lead the choice of the treatment.

Smoking cessation is the first and most important treatment for coping with COPD. If it is impossible to eliminate any other risk factors, then a strict control of the characters of life and work environment is mandatory.

General practitioners should record a complete smoking history and current status of their patients on their data bases. They are entitled to a minimal advice, which - on the existing evidences - has been shown effective and cost-effective.

If a person diagnosed as COPD is not able to quit smoking with the minimal advice - since smoking cessation is an essential therapeutic measure for these patients - then he/she is entitled to a pharmacologic and behavioural treatment (second level intervention) [1].

To-date nicotine replacement therapy (NRT) in different pharmacologic forms (patch, chewing gum, inhaler, lozanges), varenicline and slow- release bupropion (bupropion-SR) are considered first line treatment When the prescription of one of these drugs is coupled with a cognitive- behavioural treatment, a statistically significant higher percentage is granted of continuous abstinence (see Table 2).

**Table 2:** Smoking cessation therapy (Modified from MC Fiore, 2008)

**Type of intervention**	**Odds ratio (95% C.I.)**	**Abstinence rate (95% C.I.)**
Cognitive-behavioural treatment		
None	1.0	10.9
Minimal advice (<3 min)	1.3 (1.01-1.06)	13.4 (10.9-16.1)
Counseling 3–10 min	1.6 (1.2-2.0)	16.0 (12.8-19.2)
Counseling > 10 min	2.3 (2.0-2.7)	22.1 (19.4-24.7)
Pharmacologic therapy		
Placebo	1.0	13.8
Varenicline	3.1	33.2 (28.9-37.8)
Nicotine replacement therapy (NRT)		
Patch (6–14 weeks)	1.9 (1.7-2.2)	23.4 (21.3-25.8)
Chewing gum (6–14 weeks)	1.5 (1.2-1.7)	19.0 (16.5- 21.9)
Inhaler	2.1 (1.5-2.9)	24.8 (19.1-31.6)
SR Bupropion	2.0 (1.8-2.2)	24.2 (22.1-26.4)

Every Chest Physician should include smoking cessation therapy in the treatment of the smoker COPD patient. He/she should also refers the patient to a smoking cessation clinic whenever necessary [2].

There is an ongoing debate about the use of e-cigarette in a smoking cessation therapy [3]. Furthermore, encouragement is also necessary for the COPD patient to live lifestyles able to contrast sedentariness, overweight and social isolation.

#### Pharmacotherapy

It has been widely demonstrated that, in COPD patients, regular pharmacotherapy improves symptoms, lung function, and exercise tolerance [1-3]. Furthermore, regular pharmacotherapy can reduce the rate of decay of lung function [4-7], and decrease the frequency and severity of exacerbations [8-15] as well as the number and length of hospitalizations [14-20].

The main goal of the maintenance pharmacotherapy of COPD is bronchodilation. Inhaled long-acting bronchodilators are the first-line treatment for stable COPD [LABA (long-acting beta2 agonists): formoterol, salmeterol, indacaterol. LAMA (long-acting muscarinic anti-agonists): tiotropium, glycopyrronium, aclidinium].

### Recommendations

The prescription and maintenance of pharmacotherapy needs:

1. The confirmation of the diagnosis of COPD having ascertained the presence of risk factors, respiratory symptoms, and spirometric evidence of airflow obstruction.

2. An active and personalized smoking cessation program.

3. The strong recommendation for a healthier lifestyle:

• healthy nutrition program, and weight control;

• regular physical activity;

• social life.

4. Any therapeutic program must be tailored to the characteristics of the individual patient with COPD taking into account the severity of the overall clinical status on the basis of symptoms, lung function, complications, comorbidities, and, when possible, the phenotype [21].

5. In symptomatic patients with a confirmed diagnosis of COPD, dyspnea ≥ mMRC stage 1, and with pre-bronchodilator FEV_1_ ≥ 80% predicted [22] the caring physicians may consider treatment with bronchodilators [23].

6. Regular treatment with inhaled long-acting bronchodilators is recommended in symptomatic patients with a confirmed diagnosis of COPD and pre-bronchodilator FEV_1_ < 80% predicted [16,24-33].

Two clinical studies showed a better protection to exacerbations for tiotropium compared to LABA although both categories (LABA and LAMA) provided an effective bronchodilation [14-16]. Furthermore, a recent clinical study on a large population of patients has documented the clinical safety of tiotropium for the available doses and inhalers [17].

At any control visit, the followings should be evaluated:

• the adherence to the maintenance therapy;

• the changes in symptoms, and in particular in dyspnea and exercise tolerance;

• the changes in lung function: not only for FEV_1_, but also lung volumes and, when needed, DL_CO_;

• the use of rescue medications;

• the rate and severity of exacerbations;

• the frequency of hospitalizations and the length of stay;

• the adverse events.

If the patient or/and the caring physician are not satisfied with the results of the prescribed long-acting bronchodilator monotherapy, one of the followings should be considered:

• an increase in the dose of the bronchodilator according to its pharmacologic characteristics [26-29];

• the addition of a second long-acting bronchodilator with a different mechanism of action [34-44];

• the addition of inhaled corticosteroid (ICS), in patients with frequent exacerbations [8,9,11,39-41].

7 In patients with COPD, who:

• remain symptomatic despite the regular use of long acting bronchodilator(s),

• present a pre-bronchodilator FEV_1_ < 60% predicted [9], and

• suffer ≥ 2 exacerbations/year [45]

the addition of ICS to LABA may be considered^a^. The use of a single inhaler fix dose combination LABA + ICS may improve the adherence to treatment [8,9,11,46-49].

8 In those patients, the "triple therapy", i.e. LAMA + LABA + ICS, can improve lung function and quality of life, and reduce the number of hospitalizations [16,48,49].

9 In COPD patients with:

• symptoms of chronic bronchitis,

• pre-bronchodilator FEV_1_ < 50% predicted, and

• frequent exacerbations, i.e. ≥ 2/year,

the addition of a phosphodiesterase-4 inhibitor (roflumilast) on top of regular treatment with long-acting bronchodilator(s) can further improve lung function and reduce the exacerbation rate [50-54].

Conventionally, the airflow obstruction is defined as severe in COPD patients with FEV_1_ < 50% predicted and very severe in those with FEV_1_ < 30% predicted. This classification is the result of an "expert agreement" and is not either based on the evidence from prospective studies or somehow correlated to the severity of the patient’s overall clinical status. However, for operational and communication purposes, it may be useful to suggest a conventional agreement on three stages of the severity of airflow obstruction, in patients with a FEV_1_/VC < 95° predicted [40]:

• mild: FEV_1_ ≥ 80% predicted

• moderate: FEV_1_ < 80% and ≥ 50% predicted

• severe: FEV_1_ < 50% predicted^b^

Some composite indices have been suggested to take into account non only the lung function abnormalities but also some other clinical aspects relevant for the overall patient evaluation: BODE [55-57], DOSE [58], ADO [59]. However their use in the clinical settings to assess the status and the progression of COPD as well as the effects of therapeutic strategies is limited [60].

#### Oxygen and non-pharmacological therapy

Severe COPD is commonly associated with respiratory failure, which is characterized by arterial hypoxemia (PaO_2_/FiO_2_ < 300 mmHg). Evidence suggests that chronic hypoxemia with PaO_2_values less than 55–60 mmHg, if untreated by supplemental oxygen, leads to an increase in mortality [1].

In such cases, a continuative long term oxygen therapy (LTOT) is required, for a duration of at least 15 hours [1] or, better, 18–24 hours a day [2]. Oxygen administration should be continued overnight at an average flow rate of 1–2 L/min. Oxygen flow rate should be tailored to maintain the PaO_2_ value and oxygen saturation (SaO_2_%) above 60 mmHg and 92%, respectively.

In hypercapnic patients oxygen supplementation must be provided at a low flow rate in order to avoid increases of carbon dioxide retention and respiratory acidosis (pH < 7.36) [3].

According to both national and international guidelines [4,5], LTOT is indicated in stable patients, at rest while receiving the best possible treatment, and exhibiting a PaO_2_ ≤ 55 mmHg or PaO_2_ values between 56 and 59 mmHg associated with pulmonary arterial hypertension, *cor pulmonale*, o edema of the lower limbs or hematocrit > 55% in consecutive arterial blood gas analyses obtained at an interval of at least fifteen days over a two months period [5,6].

Efficacy of oxygen at the prescribed flow rate and persistence of the indication to LTOT should be verified at intervals of 3 to 12 months after prescription [7] and, on regular basis, at least once a year or whenever required by clinical changes [5].

Patients with COPD and chronic respiratory failure having frequent exacerbations requiring hospitalization, and hypercapnia (PaCO_2_ > 45 mmHg) may benefit from non-invasive ventilation (NIV) treatment [8], initiated after evaluation by competent specialists.

In selected patients, lung function improvement throughout surgical procedures such as bullectomy or lung volume reduction either by resection of emphysematous lung parenchyma or insertion of unidirectional endobronchial valves aimed to desufflate lung parenchyma or other bronchoscopic procedures still under evaluation should be considered [9]. These procedures should be reserved for thoroughly selected patients and performed in reference centers.

COPD patients aged less than 65 years, with severe lung function and clinical impairment, FEV_1_ value less than 20% of predicted, a history of frequent hospitalizations for exacerbation and requiring LTOT, should be referred for lung transplant evaluation, which has been proven to have a positive impact on outcomes such as lung function, exercise performance and quality of life, whereas its impact on survival remains unproven [10,11].

### Rehabilitation

#### Key points

Respiratory rehabilitation (RR) is defined as "a global and evidence-based multidisciplinary intervention, aimed at patients with chronic respiratory disease in all stages of severity who report symptoms and limitation of their daily activity.

If integrated in a tailored treatment for COPD, RR has the purpose of controlling symptoms, optimizing the performance status, improving participation and reducing healthcare costs by achieving clinical improvement and/or stability.

#### Outcomes

Respiratory rehabilitation (RR) improves dyspnea, exercise performance and quality of life in COPD patients. There is minor evidence for other outcomes such as prevention of complications and exacerbations, slowing of disease progression and survival. In addition, RR seems effective in cutting healthcare costs through a reduction of emergency visits and hospitalization length. In contrast, RR has no impact on FEV_1_ decline and progressive lung function deterioration in COPD.

#### Patient selection

Accurate patient selection and program personalization are of major importance for the success of RR.

#### Contraindications

Age and disease severity do not represent contraindications to RR. Current smoking is not a contraindication provided that the rehabilitation program includes sessions aimed at smoking cessation. Main contraindications are summarized in Table 3.

**Table 3:** Main contraindications to respiratory rehabilitation

**Absolute contraindications**	**Other contraindications**
Unwillingness to participate in the program	Linguistic barriers
Poor adherence to the program	Cognitive impairment
	Socio-economic barriers
	Logistic barriers (e.g. distance from hospital)

#### Structure of the rehabilitation program

A tailored rehabilitation program comprehends both useful and mandatory activities in variable combination depending on the initial assessment, and grouped in essential or fundamental and ancillary or complementary (Table 4).

**Table 4:** Classification of rehabilitation activities

**Fundamental activities**	**Complementary activities**
Optimization of pharmacotherapy	Respiratory muscle training
Training of upper and lower limbs	Chest physiotherapy
Health education	Nutritional support
Therapeutic education	
Psychologic and psychosocial support	

#### Assessment of results

Outcomes of RR are assessed with regard to every aspect of COPD. Therefore assessment of improvement in lung function disability, and social impact of the disease are currently used. The functional assessment is of major importance at the initial evaluation to customize the RR program. Indicators and outcomes are shown in Table 5.

**Table 5:** Indicators and outcomes

**Indicators**	**Outcomes**
Lung function assessment^(m)^	Improvement of exercise tolerance
Exercise tolerance assessment^(m)^	Improvement of symptoms (dyspnea)
Dyspnea assessment^(m)^	Improvement of the quality of life (QoL)
Muscular assessment^(c)^	Increase in survival rates
Psychological assessment^(c)^	Control and rationalization of costs
Nutritional assessment^(c)^	
Quality of life assessment^(m)^	

^(m)^Mandatory; ^(c)^Complementary.

## Exacerbations

### Key points

Patients with COPD experience exacerbations during the natural course of their disease condition. Frequency and severity of exacerbations are among the factors that determine the prognosis of COPD.

COPD exacerbations are the leading cause of medical consultations, hospitalizations and death among patients with greater functional compromise. Among COPD patients, exacerbations may temporarily induce conditions of relevant physical inability, even after hospital discharge.

A recent study indicates that susceptibility to exacerbations seems to remain constant over time, both among frequent exacerbators (≥2 exacerbations per year), and infrequent exacerbators (<2 events per year), irrespective of underlying disease severity ([88-90]. Patients with COPD who experience a greater number of exacerbations may be at higher risk of a more rapid decline in respiratory function [4,6].

It is of paramount importance to prevent exacerbations and to treat events promptly at symptom onset, in order to reduce the impact of exacerbations on health status and patient quality of life.

Up to 70% of the overall costs of COPD management may be attributable to exacerbations, particularly to those that require hospitalization.

Acute exacerbations of COPD (AECOPD) are defined as an acute worsening of usual symptoms in a patient with COPD (dyspnea, cough and sputum production), over and beyond normal daily variability that requires treatment modification, i.e. a course of systemic steroids and/or antibiotics [1].

During a worsening of symptoms it is important to distinguish true exacerbations from symptoms due to other conditions such as pulmonary embolism [7,8], congestive heart failure, pneumothorax, pneumonia, costal or vertebral fracture, inappropriate drug use (sedatives, narcotics and betablockers). The most common causes of exacerbations are viral and/or bronchial infections of the tracheobronchial tree [9].

### Preventing exacerbations

Measures that may be adopted for preventing exacerbations and their efficacy are summarized in Table 6.

**Table 6:** Measures that may be adopted in preventing COPD exacerbations

**Measure**	**Efficacy**
Influenza vaccination	Documented efficacy
Long term tiotropium administration	Documented efficacy
Long term LABA administration	Documented efficacy
LABA + inhaled corticosteroid administration	Documented efficacy
LAMA + LABA + ICS	Documented efficacy
Continuation of systemic steroid therapy for a brief period after AECOPD	Documented efficacy
Respiratory rehabilitation	Documented efficacy
Smoking cessation	Documented efficacy
Polysaccharide antipneumococcal vaccination	Controversial efficacy
Antioxidant-mucoactive drugs	Controversial efficacy
Bacterial lysate	Possible efficacy

### Treatment of exacerbations

In the outpatient management of exacerbations, the first step is the additional use of short acting bronchodilators (SABA or SAMA) [10], by increasing the dose or modifying the route of administration of drugs used during the stable phase.

There is evidence regarding the efficacy of administering systemic corticosteroids during an exacerbation. It is advisable not to exceed the dose of 30–40 mg a day of prednisone for 7–14 days [11-13].

Antibiotics are particularly recommended in exacerbations where both increase in sputum volume and sputum purulence are present [14-18]. There is no demonstration that for single drugs parenteral administration is superior to the oral route.

Notwithstanding prompt institution of treatment, some patients do not respond to outpatient management and may satisfy one or more criteria for hospital admission (Table 7).

**Table 7:** Criteria for appropriate hospital admission for COPD exacerbations

Inadequate or failed response to outpatient treatment
Presence of high risk comorbidity (pneumonia, arrhythmia, congestive heart failure, diabetes, liver or renal failure) or very elderly patients
Past history of frequent exacerbations
Significant increase in dyspnea and/or onset of new signs (cyanosis, peripheral edema, arrythmias)
Significant worsening in hypoxemia
Worsening in hypercapnia/respiratory acidosis (not detectable at the patient bedside)
Mental status alterations
Lack of or unreliable family assistance
Diagnostic uncertainty

Strict adherence to these criteria is of extreme importance in order to reduce inappropriate hospital admission for COPD exacerbations. In general terms, the presence of comorbidities does not alter the treatment scheme for COPD exacerbations. Comorbidities should be treated independently. Hospital admission is justified particularly when respiratory failure develops or worsens as testified by blood gas analysis. SpO_2_ values below 92% suggest presence of hypoxemia.

In exacerbations with overt respiratory failure (PaO_2_/FiO_2_ ≤ 300 mmHg) oxygen administration is necessary to maintain pulseoxymetry (SpO_2_) ≥ 93%. Values ≥ 88% may be considered acceptable when high flow oxygen may precipitate hypercapnia [19-21].

In the presence of ventilatory failure (PaCO_2_ > 45 mmHg) and/or respiratory failure (PaO_2_/FiO_2_ ≤ 300 mmHg and PaCO_2_ > 45 mmHg) with respiratory acidosis (pH ≤ 7.35), non invasive ventilation should be considered as it has been shown to reduce mortality and the need for endotracheal intubation [19-22].

## Integrated hospital-community management of patients with severe COPD

### Hospital management of the acute phase

Patients with "severe" or "very severe" COPD who experience exacerbations should be hospitalized. Based on the severity of acute respiratory failure (ARF) [1-2] treatment should be carried out in respiratory units with different intensity of management capacity (Monitoring Unit, Respiratory Intermediate Care Unit, Respiratory Intensive Care Unit) [3-5]. When ARF is associated with multiple organ failure the patient should be admitted to an Intensive Care Unit [4,5]. At discharge, collaboration between hospital based specialists and general practitioner allows continuing assistance with the use of targeted organizing models.

The hospital discharge note is the first tool to guarantee continuing home assistance, as it should include indications on the severity of COPD, degree of functional compromise as assessed by relevant lung function parameters, presence and severity of comorbidity, use of inhaled therapy, and clinical follow up. It should also indicate whether the patient is an active smoker, and set a treatment program to favour quitting.

### Home care pathway

In the community, an integrated system is required in order to ensure adequate levels of assistance outside acute care hospital settings and rehabilitation centers [6-8]. This may be obtained through shared computer systems for the management of patients and the employment of a health team that includes - in addition to a pulmonologist and the general practitioner - other health professionals (Table 8). All professionals involved should be organized and integrated into a respiratory network evenly distributed throughout the community. The team should guarantee telematic monitoring, a second opinion service active twenty four hours a day, home pulmonologist examination, and prompt hospitalization in the presence of foreseeable clinical critical conditions.

**Table 8:** Health professionals involved in home management of patients with respiratory failure

Reference physician for Home Care
Trained nurse
Respiratory therapist for rehabilitation
Psychologist
Dietician/nutritional counsellor

### Palliative and end of life care in COPD

Palliative care should be integrated within the treatment plan for patients with COPD [9-11] and be initiated when symptoms such as dyspnea, pain, depression, anxiety and constipation are not completely controlled by standard pharmacological treatment.

The term palliation encompasses interventions aimed at preventing and relieving patient suffering through symptom control, so as to stabilize or improve quality of life.

The concept of end of life assistance is instead reserved to the terminal phase of the disease and implies "comfort" or support measures for both the sick person, and for his/her family members [10].

Palliation and end of life care require multidisciplinary involvement of physicians, nurses, physiotherapists, psychologists, social workers, home care providers, and clericals when requested [11].

### Telemedicine and teleassistance

The management of chronic conditions and continuing assistance may be greatly improved through the application of innovative technologies, among which telemedicine, teleassistance and more in general Information and Communication Technology (ICT). Application of these systems is particularly useful in guaranteeing a network-based operative frame for taking charge of patients with chronic disease. The National Program for Research and Formation in Telemedicine [12] indicates telemedicine as "a particular means of providing health assistance from community-based institutions, that allows integrated delivery of diagnostic and management medical measures, overcoming the barriers associated with territorial distribution of different competences, bridging the gap between subscribers and the experts, and reducing temporal fragmentation of interventions on single patients".

The use of telemedicine tools is aimed at reaching a greater degree of interaction between the community and reference clinical centers, reducing the need for transferral of frail and often elderly patients. Telemedicine guarantees contacts between centres with different clinical expertise, dialogue through equipment present in the patient’s home, assistance to remote or isolated areas, emergency interventions, solidarity to low income countries. The Italian National Health Plan for the years 2011–2013 [13] underlines the need for telemedicine implementation in order to guarantee access to specific health assistance. Tables 9 and 10 respectively summarize the aims and critical issues of teleassistance.

**Table 9:** Aims of teleassistance

Improve patient quality of life
Improve family member’s quality of life
Increase the degree of patients safety at home
Avoid hospitalizations
Reduce outpatient general practitioner consultations
Reduce outpatient respiratory specialist consultation
Reduce need for patient transferral, and associated costs

**Table 10:** Critical aspects in teleassistance

Possible loss of direct patient-physician contact
Personal data
Difficulties in accessing the assistance web
Poor interactivity between computer systems
Paucity of uniform political strategies across the nation
Paucity of definitive data on the efficacy of the system
Absence of specific legislation on the aspects of security regarding both the patient and the prescribing physician

### The role of institutions

In consideration of organisational and institutional competences, it is helpful that central institutions (which are in charge) do ensure the training of an appropriate number of Specialists for the needs of assistance. Furthermore, in consideration of the epidemiological data, the Ministry of Health and Regional Health Institutions do insert a specific section for acute and chronic respiratory diseases in their planning, namely for COPD; the Regions and Local Health Units do their best for the reinforcement and homogeneity of the network for lung function assessment. At the same time it is helpful that in the whole country the distribution of Pulmonary Divisions with Units of Respiratory Intensive Care or Intermediate Intensive Units or Respiratory Monitoring Units is organized according to precise criteria of inhabitants number and/or extension of the area. In addition it should be realized, at least at regional level, a telemonitoring service active twenty four hours a day through a call center, which can telematically receive all the parameters that should be monitored (pneumological teleassistance), and at the same time can guarantee a comprehensive healthcare support to the patient with respiratory failure. Furthermore, it is helpful that in every region some rehabilitation centers for post-acute patients can be found, with a ratio of day-beds suitable for population; at the same time, centers of outpatient respiratory rehabilitation can be active in every Local Health Unit and able to give the care needed by the patient in the steady phase of the disease, with costs under control. Last, it is important the Ministry of Health includes the therapeutical education even in the LEAs (Essential Levels of Healthcare) of COPD patient.

## Endnotes

^a^EMA–AIFA for salmeterol 50/fluticasone 500 mcg bid "symptomatic treatment of COPD patients with FEV_1_ < 60% predicted (pre-bronchodilatar) and a clinical history of frequent exacerbations, with important symptoms notwithstanding the regular therapy with bronchodilators".

^b^It must be considered as pre-bronchodilator.

## Appendix

References regarding paragraph "Introduction" and "Methodological note"

1. Warsi A, Wang PS, LaValley MP, Avorn J, Solomon DH: **Self management education programs in chronic disease: a systematic review and methodological critique of the literature.***Arch Intern Med* 2004, **164:**1641–1649.

2. WHO Working Group Report: *Therapeutic Patient Education: Continuing education programmes for healthcare providers in the field of prevention of chronic diseases.* Copenhagen: WHO Regional Office for Europe; 1998.

3. Ministero Salute: *Quaderno "Appropriatezza clinica, strutturale, tecnologica e operativa per la prevenzione, diagnosi e terapia dell’obesità e del diabete mellito"*.

References regarding paragraph "COPD Definition and diagnosis"

1. Hogg JC, Macklem PT, Thurlbeck WM: **Site and nature of airway obstruction in chronic obstructive lung disease.***N Engl J Med* 1968, **278:**1355–1360.

2. Cosio M, Ghezzo H, Hogg JC, Corbin R, Loveland M, Dosman J, Macklem PT: **The relations between structural changes in small airways and pulmonary-function tests.***N Engl J Med* 1978, **298:**1277–1281.

3. Hogg JC, Chu F, Utokaparch S, Woods R, Elliott WM, Buzatu L, Cherniack RM, Rogers RM, Sciurba FC, Coxson HO, Paré PD: **The nature of small-airway obstruction in chronic obstructive pulmonary disease.***N Eng J Med* 2004, **350:**2645–2653.

4. Barnes PJ: **Small airways in COPD.***N Engl J Med* 2004, **350:**2635–2637.

5. Celli BR, Halbert RJ, Enright P, Brusasco V: **Should we abandon FEV**_**1**_**/FVC < 0.70 to detect airway obstruction? No/Yes.***Chest* 2010, **138:**1037–1042.

6. Sorino C, Battaglia S, Scichilone N, Pedone C, Antonelli-Incalzi R, Sherrill D, Bellia V: **Diagnosis of airway obstruction in the elderly: contribution of the SARA study.***Int J COPD* 2012, **7:**389–395.

7. Mannino DM, Diaz-Guzman E: **Interpreting lung function data using 80% predicted and fixed thresholds identifies patients at increate risk of mortality.***Chest* 2012, **141:**73–80.

8. O’Donnell DE, Aaron S, Bourbeau J, Hernandez P, Marciniuk DD, Balter M, Ford G, Gervais A, Goldstein R, Hodder R, Kaplan A, Keenan S, Lacasse Y, Maltais F, Road J, Rocker G, Sin D, Sinuff T, Voduc N: **Canadian Thoracic Society recommendation for management of chronic obstructive pulmonary disease - 2007 update.***Can Respir J* 2007, **14:**5b–32b.

9. Saetta M, Ghezzo H, Kim WD, King M, Angus GE, Wang NS, Cosio MG: **Loss of alveolar attachments in smokers. A morphometric correlate of lung function impairment.***Am Rev Respir Dis* 1985, **132:**894–900.

10. Siafakas NM, Vermeire P, Pride NB, Paoletti P, Gibson J, Howard P, Yernault JC, Decramer M, Higenbottam T, Postma DS, Rees PJ, on behalf of the Task Force*:***Optimal assessment and management of chronic obstructive pulmonary disease. ERS, consensus statement.***Eur Respir J* 1995, **8:**1398–1420.

**References regarding paragraph** "**Management of COPD in stable state"**

1. Fiore MC: *Treating tobacco use and dependance: 2008 Update.* US Department of Health and Human Services, Ministerodella Salute. Istituto Superiore di Sanità. Linee guida cliniche per promuovere la cessazione dell’abitudine al fumo. Aggiornamento 2008.www.iss.it/ofad.

2. Tønnesen P, Carrozzi L, Fagerström KO, Gratziou C, Jimenez-Ruiz C, Nardini S, Viegi G, Lazzaro C, Campell IA, Dagli E, West R: **Smoking cessation in patients with respiratory diseases: a high priority, integral component of therapy.***Eur Respir J* 2007, **29:**390–417.

3. Implicazioni relative alla salute derivanti dall’uso della sigaretta elettronica: *Documento di posizione congiunto dell’Associazione Italiana Pneumologi Ospedalieri (AIPO) e della Società Italiana di Medicina Respiratoria (SIMeR).* Aprile 2013.www.aiponet.it; www.simernet.it.

References regarding paragraph "Pharmacotherapy"

1. Centre NCG: *Chronic Obstructive Pulmonary Disease: Management of Chronic Obstructive Pulmonary Disease in Adults in Primary and Secondary Care.* London: National Clinical Guideline Centre; 2010. Available from: http://guidance.nice.org.uk/CG101/Guidance/pdf/English.

2. Qaseem A, Wilt TJ, Weinberger SE, Hanania NA, Criner G, van der Molen T, Marciniuk DD, Denberg T, Schünemann H, Wedzicha W, MacDonald R, Shekelle P; American College of Physicians; American College of Chest Physicians; American Thoracic Society; European Respiratory Society: **Diagnosis and management of stable chronic obstructive pulmonary disease: a clinical practice guideline from the ACP, ACCP, ATS and ERS.***Ann Intern Med* 2011, **155:**179–191.

3. Vestbo J, Hurd SS, Agustì AA, Jones PW, Vogelmeier C, Anzueto A, Barnes PJ, Fabbri LM, Martinez FJ, Nishimura M, Stockley RA, Sin DD, Rodriguez-Roisin R: **Global strategy for the diagnosis, management, and prevention of chronic obstructive pulmonary disease. GOLD executive summary.***Am J Respir Crit Care Med* 2013, **187:**347–365.

4. Celli BR, Thomas NE, Anderson JA, Ferguson GT, Jenkins CR, Jones PW, Vestbo J, Knobil K, Yates JC, Calverley PM: **Effect of pharmacotherapy on rate of decline of lung function in chronic obstructive pulmonary disease.***Am J Respir Crit Care Med* 2008, **178:**332–338.

5. Decramer M, Celli B, Kesten S, Lystig T, Mehra S, Tashkin DP, for the UPLIFT Investigators: **Effect of tiotropium on outcomes in patients with moderate chronic obstructive pulmonary disease (UPLIFT): a prespecified subgroup analysis of a randomized controlled trial.***Lancet* 2009, **374:**1171–1178.

6. Troosters T, Celli B, Lystig T, Kesten S, Mehra S, Tashkin DP, Decramer M; Uplift Investigators: **Tiotropium as a first maintenance drug in COPD: secondary analysis of the UPLIFT trial.***Eur Respir J* 2010, **36:**65–73.

7. Vestbo J, Edwards LD, Scanlon PD, Yates JC, Agusti A, Bakke P, Calverley PM, Celli B, Coxson HO, Crim C, Lomas DA, MacNee W, Miller BE, Silverman EK, Tal-Singer R, Wouters E, Rennard SI; ECLIPSE Investigators: **Changes in forced expiratory volume in 1 second over time in COPD.***N Engl J Med* 2011, **365:**1184–1192.

8. Calverley PMA, Pauwels R, Vestbo J, Jones P, Pride N, Gulsvik A, Anderson J, Maden C; TRial of Inhaled STeroidsANd long-acting beta2 agonists study group: **Combined salmeterol and fluticasone in the treatment of chronic obstructive pulmonary disease: a randomized controlled trial.***Lancet* 2003, **361:**449–456.

9. Calverley PMA, Anderson JA, Celli B, Ferguson GT, Jenkins C, Jones PW, Yates JC, Vestbo J; TORCH investigators: **Salmeterol and fluticasone propionate and survival in chronic obstructive pulmonary disease.***N Engl J Med* 2007, **356:**775–789.

10. Tashkin DP, Celli B, Senn S, Burkhart D, Kesten S, Menjoge S, Decramer M; UPLIFT Study Investigators:**A 4-year trial of tiotropium in chronic obstructive pulmonary disease.***N Engl J Med* 2008, **359:**1543–1554.

11. Jenkins CR, Jones PW, Calverley PM, Celli B, Anderson JA, Ferguson GT, Yates JC, Willits LR, Vestbo J: **Efficacy of salmeterol/fluticasone propionate by GOLD stage of chronic obstructive pulmonary disease: analysis from the randomized, placebo-controlled TORCH study.***Respir Res* 2009, **10:**59–68.

12. Wedzicha JA, Calverley PMA, Seemugal TA, Hagan G, Ansari Z, Stockley RA, for the INSPIRE Investigators: **The prevention of chronic obstructive pulmonary disease exacerbations by salmeterol/fluticasone propionate or tiotropium bromide.***Am J Respir Crit Care Med* 2008, **177:**19–26.

13. Kardos P, Wencker M, Glaab T, Vogelmeier C: **Impact of salmeterol/fluticasone propionate versus salmeterol on exacerbations in severe chronic obstructive pulmonary disease.***Am J Respir Crit Care Med* 2007, **175:**144–149.

14. Vogelmeier C, Hederer B, Glaab T, Schmidt H, Rutten-van Mölken MP, Beeh KM, Rabe KF, Fabbri LM; POET-COPD Investigators: **Tiotropium versus salmeterol for the prevention of exacerbations of COPD**. *N Eng J Med* 2011, **364:**1093–1103.

15. Decramer M, Chapman KR, Dahl R, Frith P, Devouassoux G, Fritscher C, Cameron R, Shoaib M, Lawrence D, Young D, McBryan D; INVIGORATE investigators: **Once-daily indacaterol versus tiotropium for patients with severe chronic obstructive pulmonary disease (INVIGORATE): a randomised, blinded, parallel-group study.***LancetRespir Med* 2013, **1**(7)**:**524–533.

16. Wedzicha JA: **Choice of bronchodilator therapy for patients with COPD.***N Engl J Med* 2011, **364:**1167–1168.

17. Wise RA, Anzueto A, Cotton D, Dahl R, Devins T, Disse B, Dusser D, Joseph E, Kattenbeck S, Koenen-Bergmann M, Pledger G, Calverley P; TIOSPIR Investigators: **Tiotropium Respimat inhaler and the risk of death in COPD.***N Eng J Med* 2013, **369:**1491–1501.

18. Kerwin E, Hébert J, Gallagher N, Martin C, Overend T, Alagappan VK, Lu Y, Banerji D: **Efficacy and safety of NVA237 versus placebo and tiotropium in patients with COPD: the GLOW2 study.***Eur Respir J* 2012, **40:**1106–1114.

19. Aaron SD, Vandemheen KL, Fergusson D, Maltais F, Bourbeau J, Goldstein R, Balter M, O’Donnell D, McIvor A, Sharma S, Bishop G, Anthony J, Cowie R, Field S, Hirsch A, Hernandez P, Rivington R, Road J, Hoffstein V, Hodder R, Marciniuk D, McCormack D, Fox G, Cox G, Prins HB, Ford G, Bleskie D, Doucette S, Mayers I, Chapman K, Zamel N, FitzGerald M; Canadian Thoracic Society/Canadian Respiratory Clinical Research Consortium: **Tiotropium in combination with placebo, salmeterol, or fluticasone-salmeterol for treatment of chronic obstructive pulmonary disease. A randomized trial.***Ann Intern Med* 2007, **146:**545–555.

20. Short PM, Williamson PA, Elder DHJ, Lipworth SI, Schembri S, Lipworth BJ: **The impact of tiotropium on mortality and exacerbations when added to inhaled corticosteroids and long-acting β-agonist therapy in COPD.***Chest* 2012, **141:**81–86.

21. Camicilotti G, Bigazzi F, Paoletti M, Cestelli L, Lavorini F, Pistolesi M: **Pulmonary function and sputum characteristics predict CT phenotype and severity of COPD.***Eur Respir J* 2013, **42:**626–635.

22. Ofir D, Laveneziana P, Webb KA, Lam YM, O’Donnel DE: **Mechanisms of dyspnea during cycle exercise in symptomatic patients with GOLD stage I chronic obstructive pulmonary disease.***Am J Respir Crit Care Med* 2008, **177:**622–629.

23. O’Donnell DE, Laveneziana P, Ora J, Webb KA, Lam YM, Ofir D: **Evaluation of acute bronchodilator reversibility in patients with symptoms of GOLD stage I COPD.***Thorax* 2009, **64:**216–223.

24. Tashkin DP, Fabbri LM: **Long-acting beta-agonists in the management of chronic obstructive pulmonary disease: current and future agents.***Respir Res* 2010, **11:**149.

25. Rossi A, Khirani S, Cazzola M: **Long-acting β2-agonist (LABA) in chronic obstructive pulmonary disease: efficacy and safety.***Int J COPD* 2008, **3:**521–529.

26. Jones PW, Donohue JF, Nedelman J, Pascoe S, Pinaul G, Lassen C: **Correlating changes in lung function with patient outcomes in chronic obstructive pulmonary disease: a pooled analysis.***Respir Res* 2011, **12:**161.

27. Dahl R, Greefhorst LA, Nowak D, Nonikov V, Byrne AM, Thomson MH, Till D, Della Cioppa G; Formoterol in Chronic Obstructive Pulmonary Disease I Study Group: **Inhaled formoterol dry powder versus ipratropium bromide in chronic obstructive pulmonary disease study group.***Am J RespirCrit Care Med* 2001, **164:**778–784.

28. Rossi A, Kristufek P, Levine BE, Thomson MH, Till D, Kottakis J, Della Cioppa G; Formoterol in Chronic Obstructive Pulmonary Disease (FICOPD) II Study Group: **Comparison of the efficacy, tolerability, and safety of formoterol dry powder and oral, slow release theophylline in the treatment of COPD.***Chest* 2002, **121:**1058–1069.

29. Rossi A, Polese G: **Indacaterol: a comprehensive review.***Int J COPD* 2013, **8:**353–363.

30. Jones P, Singh D, Bateman ED, Agusti A, Lamarca R, de Miquel G, Segarra R, Caracta C, Garcia Gil E: **Efficacy and safety of aclidinium bromide administered twice a day in patients with COPD; the ATTAIN study.***EurRespir J* 2012, **40:**830–836.

31. Cazzola M, Page CP, Matera MG: **Aclidinium bromide for the treatment of chronic obstructive pulmonary disease.***ExpOpinPharmacother* 2013, **14:**1205–1214.

32. Beier J, Kirsten AM, Mroz R, Segarra R, Chuecos F, Caracta C, Gil EG: **Efficacy and safety of aclidinium bromide compared with placebo and tiotropium in patients with moderate-to-severe chronic obstructive pulmonary disease: results from a 6-week, randomized, controlled phase IIIb study.***COPD* 2013, **10:**511–522.

33. Buhl R, Banerji D: **Prophile of Glycopyrronium for once-daily treatment of moderate-to-severe COPD.***Int J COPD* 2012, **2:**729–741.

34. COMBIVENT Inhalation Aerosol Study Group: **In chronic obstructive pulmonary disease, a combination of ipratropium and alburerol is more effective than either agent alone. An 85-day multicenter trial.***Chest* 1994, **105:**1411–1419.

35. Benayoun S, Ernst P, Suissa S: **The impact of combined inhaled bronchodilator therapy in the treatment of COPD.***Chest* 2001, **119:**85–92.

36. van Noord JA, Aumann JL, Janssens E, Verhaert J, Smeets JJ, Mueller A, Cornelissen PJ: **Effects of tiotropium with and without formoterol on airflow obstruction and resting hyperinflation in patients with COPD.***Chest* 2006, **129:**509–517.

37. Rabe KF, Timmer W, Sagkriotis A, Viel K: **Comparison of a combination of tiotropium plus formoterol to salmeterol plus fluticasone in moderate COPD.***Chest* 2008, **134:**255–262.

38. van Noord JA, Buhl R, Laforce C, Martin C, Jones F, Dolker M, Overend T: **QVA149 demonstrates superior bronchodilation compared with indacaterol or placebo in patients with chronic obstructive pulmonary disease.***Thorax* 2010, **65:**1086–1091.

39. Karner C, Cates CJ: **Long-acting beta2-agonist in addition to tiotropium versus either tiotropium or long-acting beta2-agonist alone for chronic obstructive pulmonary disease.***Cochrane Database Syst Rev* 2012, **4**, CD008989.

40. Mahler DA, D’Urzo A, Bateman ED, Ozkan SA, White T, Peckitt C, Lassen C, Kramer B; INTRUST-1 and INTRUST-2 study investigators: **Concurrent use of indacaterol plus tiotropium in patients with COPD provides superior bronchodilation compared with tiotropium alone: a randomized, double-blind comparison.***Thorax* 2012, **67:**781–788.

41. Tashkin DP, Ferguson GT: **Combination bronchodilator in the management of chronic obstructive pulmonary disease.***Respir Res* 2013, **14:**49.

42. Vogelmeier CF, Bateman ED, Pallante J, Alagappan VK, D’Andrea P, Chen H, Banerji D: **Efficacy and safety of once-daily QVA149 compared with twice-daily salmeterol-fluticasone in patients with chronic obstructive pulmonary disease (ILLUMINATE): a randomised, double-blind, parallel group study.***Lancet Respir Med* 2013, **1:**51–60.

43. Wedzicha JA, Decramer M, Ficker JH, Niewoehner DE, Sandström T, Taylor AF, D’Andrea P, Arrasate C, Chen H, Banerji D: **Analysis of chronic obstructive pulmonary disease exacerbations with the dual bronchodilator QVA149 compared with glycopyrronium and tiotropium (SPARK): a randomised, double-blind, parallel-group study.***Lancet Respir Med* 2013, **1:**199–209.

44. Bateman ED, Ferguson GT, Barnes N, Gallagher N, Green Y, Henley M, Banerji D: **Dual bronchodilation with QVA149 versus single bronchodilator therapy: the SHINE study.***Eur Respir J* 2013, **42:**1484–1494.

45. Hurst JR, Vestbo J, Anzueto A, Locantore N, Müllerova H, Tal-Singer R, Miller B, Lomas DA, Agusti A, Macnee W, Calverley P, Rennard S, Wouters EF, Wedzicha JA; Evaluation of COPD Longitudinally to Identify Predictive Surrogate Endpoints (ECLIPSE) Investigators: **Susceptibility to exacerbation in chronic obstructive pulmonary disease.***N Engl J Med* 2010, **363:**1128–1138.

46. Szafranski W, Cukier A, Ramirez A, Menga G, Sansores R, Nahabedian S, Peterson S, Olsson H: **Efficacy and safety of budesonide/formoterol in the management of chronic obstructive pulmonary disease.***Eur Respir J* 2003, **21:**74–81.

47. Calverley PM, Boonsawat W, Cseke Z, Zhong N, Peterson S, Olsson H: **Maintenance therapy with budesonide and formoterol in chronic obstructive pulmonary disease.***Eur Respir J* 2003, **22:**912–919.

48. Short PM, Williamson PA, Elder DH, Lipworth SI, Schembri S, Lipworth BJ: **The impact of tiotropium on mortality and exacerbations when added to inhaled corticosteroids and long-acting - agonist therapy in COPD.***Chest* 2012, **141:**81–86.

49. Welte T, Miravitlles M, Hernandez P, Eriksson G, Peterson S, Polanowski T, Kessler R: **Efficacy and tolerability of budesonide/formoterol added to tiotropium in patients with chronic obstructive pulmonary disease.***Am J Respir Crit Care Med* 2009, **180:**741–750.

50. Rabe KF, Bateman ED, O’Donnell D, Witte S, Bredenbröker D, Bethke TD: **Roflumilast –an oral anti-inflammatory treatment for chronic obstructive pulmonary disease: a randomized controlled trial.***Lancet* 2005, **366:**563–571.

51. Calverley PMA, Sanchez-Toril F, Mclvor A, Teichmann P, Bredenbröker D, Fabbri LM: **Effect of 1-year treatment with roflumilast in severe chronic obstructive pulmonary disease.***Am J Respir Crit Care Med* 2007, **176:**154–161.

52. Calverley PMA, Rabe KF, Goehring U-M, Kristiansen S, Fabbri LM, Martinez F-J, for the M2-124 and M2-125 study groups: **Roflumilast in symptomatic chronic obstructive pulmonary disease: two randomised clinical trials.***Lancet* 2009, **374:**685–694.

53. Fabbri LM, Calverley PM, Izquierdo-Alonso JL, Bundschuh DS, Brose M, Martinez FJ, Rabe KF; M2-127 and M2-128 study groups: **Roflumilast in moderate-to-severe chronic obstructive pulmonary disease treated with long acting bronchodilators: two randomised clinical trials.***Lancet* 2009, **374:**695–703.

54. Beghè B, Rabe KF, Fabbri LM: **Phosphodiesterase-4 inhibitor therapy for lung diseases.***Am J Respir Crit Care Med* 2013, **188:**271–278.

55. Celli BR, Cote CG, Marin JM, Casanova C, Montes de Oca M, Mendez RA, Pinto Plata V, Cabral HJ: **The body-mass index, airflow obstruction, dyspnea, and exercise capacity index in chronic obstructive pulmonary disease.***N Engl J Med* 2004, **350:**1005–1012.

56. Celli BR, Calverley PM, Rennard SI, Wouters EF, Agusti A, Anthonisen N, Macnee W, Jones P, Pride N, Rodriguez-Roisin R, Rossi A, Wanner A: **Proposal for a multidimensional staging system for chronic obstructive pulmonary disease.***Respir Med* 2005, **99:**1546–1554.

57. Funk GC, Kirchheiner K, Burghuber OC, Hartl S: **BODE index versus GOLD classification for explaining anxious and depressive symptoms in patients with COPD – a cross-sectional study.***Respir Res* 2009, **10:**1.

58. Jones RC, Donaldson GC, Chavannes NH, Kida K, Dickson-Spillmann M, Harding S, Wedzicha JA, Price D, Hyland ME: **Derivation and validation of a composite index of severity in chronic obstructive pulmonary diseases.***Am J Respir Crit Care Med* 2009, **180:**1189–1195.

59. Puhan MA, Garcia-Aymerich J, Frey M, terRiet G, Antó JM, Agustí AG, Gómez FP, Rodríguez-Roisín R, Moons KG, Kessels AG, Held U: **Expansion of the prognostic assessment of patients with chronic obstructive pulmonary disease: the updated BODE index and the ADO index.***Lancet* 2009, **374:**704–711.

60. Rossi A, Zanardi E: **E pluribus plurima: Multidimensional indices and clinical phenotypes in COPD.***Respir Res* 2011, **12:**15.

References regarding paragraph "Oxygen and non-pharmacological therapy"

1. **Long term domiciliary oxygen therapy in chronic hypoxic corpulmonale complicating chronic bronchitis and emphysema. Report of the Medical Research Council Working Party.***Lancet* 1981, **1:**681–686.

2. Nocturnal Oxygen Therapy Trial Group: **Continuous or nocturnal oxygen therapy in hypoxemic chronic obstructive lung disease: a clinical trial.***Ann Intern Med* 1980, **93:**391–398.

3. Plant PK, Owen JL, Elliott MW: **One year period prevalence study of respiratory acidosis in acute exacerbations of COPD: implications for the provision of non-invasive ventilation and oxygen administration.***Thorax* 2000, **55:**550–554.

4. Corrado A, Renda T, Bertini S: **Long-Term Oxygen Therapy in COPD: evidences and open questions of current indications.***MonaldiArchChestDis* 2010, **73:**34–43.

5. *Linee Guida "Insufficienza Respiratoria" Regione Toscana.* 2010.

6. **Linee Guida per la Ossigenoterapia a lungo termine (OTLT). Aggiornamento anno 2004.***Rassegna di Patologia dell’Apparato Respiratorio* 2004, **19:**206–219.

7. Guyatt GH, Nonoyama M, Lacchetti C, Goeree R, McKim D, Heels-Ansdell D, Goldstein R: **A randomized trial of strategies for assessing eligibility for long-term domiciliary oxygen therapy.***Am J Respir Crit Care Med* 2005, **172:**573–580.

8. Clini E, Sturani C, Rossi A, Viaggi S, Corrado A, Donner CF, Ambrosino N; Rehabilitation and Chronic Care Study Group, Italian Association of Hospital Pulmonologists (AIPO): **The Italian multicentre study on noninvasive ventilation in chronic obstructive pulmonary disease patients.***Eur Respir J* 2002, **20:**529–538.

9. Criner GJ: **Lung volume reduction as an alternative to transplantation for COPD.***Clin Chest Med* 2011, **32:**379–397.

10. Garrity ER, Moore J, Mulligan MS, Shearon TH, Zucker MJ, Murray S: **Heart and lung transplantation in the United States, 1996–2005.***Am J Transplant* 2007, **7:**1390–1403.

11. Cai J: **Double- and single-lung transplantation: an analysis of twenty years of OPTIN/UNOS registry data.***ClinTranspl* 2007**:**1–8.

Guidelines: General bibliography regarding paragraph "Rehabilitation"

Guidelines

ACCP/AACVPR: **Pulmonary rehabilitation. Joint ACCP/AACVPR evidence-based clinical practice guidelines.***Chest* 2007, **131:**4–42.

ATS/ERS Task Force: *Standards for the Diagnosis and Treatment of Patients with COPD.* 2004. available on line, www.ers-education.org.

British Thoracic Society: **Guidelines for the physiotherapy management of the adult medical, spontaneously breathing patients.***Thorax* 2009, **64**(suppl)**:**1–51.

British Thoracic Society: **Pulmonary rehabilitation.***Thorax* 2001, **56:**827–834.

Statements – Consensus – Position Papers

Ambrosino N, Vitacca M, Rampulla C: **Percorsi riabilitativi nelle malattie respiratorie.***Fondazione Maugeri IRCCS "I Documenti"* 1997, (n°11).

Nici L, Donner C, Wouters E, Zuwallack R, Ambrosino N, Bourbeau J, Carone M, Celli B, Engelen M, Fahy B, Garvey C, Goldstein R, Gosselink R, Lareau S, MacIntyre N, Maltais F, Morgan M, O’Donnell D, Prefault C, Reardon J, Rochester C, Schols A, Singh S, Troosters T; ATS/ERS Pulmonary Rehabilitation Writing Committee: American Thoracic Society/European Respiratory Society: **Statement on pulmonary rehabilitation.***Am J Respir Crit Care Med* 2006, **173:**1390–1413.

Associazione Italiana Pneumologi Ospedalieri: **Raccomandazioni sulla Riabilitazione Respiratoria.***RassPatol App Respir* 2007, **22:**264–288.

European Society of Intensive Care Medicine: **Physiotherapy for adult patients with critical illness: recommendations of ERS and ESICM Task Force on Physiotherapy for critically ill patients.***Intensive Care Medicine* 2008, **34:**1188–1199.

California pulmonary Rehabilitation Collaborative Group: **Effects of pulmonary rehabilitation on dyspnea, quality of life, and healthcare costs in California.***J CardiopulmRehabil* 2004, **24:**52–62.

Revisions – Meta-analyses

Casaburi R, ZuWallack R: **Pulmonary rehabilitation for management of chronic obstructive pulmonary disease.***N Engl J Med* 2009, **360:**1329–1335.

Nici L, Raskin J, Rochester CL, Bourbeau JC, Carlin BW, Casaburi R, Celli BR, Cote C, Crouch RH, Diez-Morales LF, Donner CF, Fahy BF, Garvey C, Goldstein R, Lane-Reticker A, Lareau SC, Make B, Maltais F, McCormick J, Morgan MD, Ries A, Troosters T, ZuWallack R: **Pulmonary rehabilitation: what we know and what we need to know.***J CardiopulmRehabilPrev* 2009, **29:**141–151.

Lacasse Y, Goldstein R, Lasserson TJ, Martin S: **Pulmonary rehabilitation for chronic obstructive pulmonary disease.***Cochrane Database Syst Rev* 2006, **4**, CD003793.

Puhan M, Scharplatz M, Troosters T, Walters EH, Steurer J: **Pulmonary rehabilitation following exacerbations of chronic obstructive pulmonary disease.***Cochrane Database Syst Rev* 2009, **1**, CD005305.

Troosters T, Casaburi R, Gosselink R, Decramer M: **Pulmonary rehabilitation in chronic obstructive pulmonary disease.***Am J Respir Crit Care Med* 2005, **172:**19–38.

Troosters T, Gosselink R, Janssens W, Decramer M: **Exercise training and pulmonary rehabilitation: new insights and remaining challenges.***Eur Respir Rev* 2010, **19**(115)**:**24–29.

References regarding paragraph "Exacerbations"

1. Hurst JR, Vestbo J, Anzueto A, Locantore N, Müllerova H, Tal-Singer R, Miller B, Lomas DA, Agusti A, Macnee W, Calverley P, Rennard S, Wouters EF, Wedzicha JA; Evaluation of COPD Longitudinally to Identify Predictive Surrogate Endpoints (ECLIPSE) Investigators: **Susceptibility to exacerbation in chronic obstructive pulmonary disease.***N Engl J Med* 2010, **363:**1128–1138.

2. Trappenburg JCA, van Deventer AC, Troosters T, Verheij TJ, Schrijvers AJ, Lammers JW, Monninkhof EM: **The impact of using different symptom-based exacerbation algorithms in patients with COPD.***Eur Respir J* 2011, **37:**1260–1268.

3. Mackay AL, Donaldson GC, Patel ARC, Jones PW, Hurst JR, Wedzicha JA: **Usefulness of the chronic obstructive pulmonary disease assessment test to evaluate severity of COPD exacerbations.***Am J Respir Crit Care Med* 2012, **185:**1218–1224.

4. Seemugal TAR, Donaldson GC, Bhowmik A, Jeffries DJ, Wedzicha JA: **Time course and recovery of exacerbations in patients with chronic obstructive pulmonary disease.***Am J Respir Crit Care Med* 2000, **161:**1608–1613.

5. Donaldson GC, Seemugal TAR, Bhowmik A, Wedzicha A: **Relationship between exacerbation frequency and lung function decline in chronic obstructive pulmonary disease.***Thorax* 2002, **57:**847–852.

6. Anzueto A, Leimer I, Kesten S: **Impact of frequency of COPD exacerbations on pulmonary function, health status and clinical outcomes.***Int J COPD* 2009, **4:**245–251.

7. Tillie-Leblond I, Marquette CH, Perez T, Scherpereel A, Zanetti C, Tonnel AB, Remy-Jardin M: **Pulmonary embolism in patients with unexplained exacerbation of chronic obstructive pulmonary disease: prevalence and risk factors.***Ann Intern Med* 2006, **144:**390–396.

8. Rizkallah J, Man SFP, Sin DD: **Prevalence of pulmonary embolism in acute exacerbations of COPD. A systematic review and meta-analysis.***Chest* 2009, **135:**786–793.

9. Papi A, Bellettato CM, Braccioni F, Romagnoli M, Casolari P, Caramori G, Fabbri LM, Johnston SL: **Infections and airway inflammation in chronic obstructive pulmonary disease severe exacerbations.***Am J Respir Crit Care Med* 2006, **173:**1114–1121.

10. Di Marco F, Verga M, Santus P, Morelli N, Cazzola M, Centanni S: **Effect of formoteroltiotropium and their combination in patients with acute exacerbation of chronic obstructive pulmonary disease. A pilot study.***Respir Med* 2006, **100:**1925–1932.

11. Albert RK, Martin TR, Lewis SW: **Controlled clinical trial on methylprednisolone in patients with chronic bronchitis and acute respiratory insufficiency.***Ann Intern Med* 1980, **92:**753–758.

12. Davies L, Angus RM, Calverley PM: **Oral corticosteroids in patients admitted to hospital with exacerbations of chronic obstructive pulmonary disease: a prospective randomized controlled trial.***Lancet* 1999, **354:**456–460.

13. Niewoehner DE, Erbland ML, Deupree RH, Collins D, Gross NJ, Light RW, Anderson P, Morgan NA: **Effect of systemic glucocorticoids on exacerbations of chronic obstructive pulmonary disease. Department of Veterans Affairs Cooperative Study Group.***N Engl J Med* 1999, **340:**1941–1947.

14. Anthonisen NR, Manfreda J, Warren CP, Hershfield ES, Harding GK, Nelson NA: **Antibiotic therapy in exacerbations of chronic obstructive pulmonary disease.***Ann Intern Med* 1987, **106:**196–204.

15. Saint S, Bent S, Vittinghoff E, Grady D: **Antibiotics in chronic obstructive pulmonary disease exacerbations. A meta-analysis.***JAMA* 1995, **273:**957–960.

16. Stockley RA, O’Brien C, Pye A, Hill SL: **Relationship of sputum color to nature and outpatient management of acute exacerbations of COPD.***Chest* 2000, **117:**1638–1645.

17. Roede BM, Bresser P, Prins JM, Schellevis F, Verheij TJ, Bindels PJ: **Reduced risk of next exacerbation and mortality associated with antibiotic use in COPD.***Eur Respir J* 2009, **33:**282–288.

18. Daniels JMA, Snijders D, de Graaff CS, Vlaspolder F, Jansen HM, Boersma WJ: **Antibiotics in addition to systemic corticosteroids for acute exacerbations of chronic obstructive pulmonary disease.***Am J Respir Crit Care Med* 2012, **181:**150–157.

19. Celli BR, MacNee W, and committee members: **Standards for the diagnosis and treatment of patients with COPD: a summary of the ATS/ERS position paper.***Eur Respir J* 2004, **23:**932–946.

20. Siafakas NM, Vermeire P, Pride NB, Paoletti P, Gibson J, Howard P, Yernault JC, Decramer M, Higenbottam T, Postma DS, et al: **Optimal assessment and management of chronic obstructive pulmonary disease. ERS, consensus statement.***Eur Respir J* 1995, **8:**1398–1420.

21. O’Donnell DE, Aaron S, Bourbeau J, Hernandez P, Marciniuk DD, Balter M, Ford G, Gervais A, Goldstein R, Hodder R, Kaplan A, Keenan S, Lacasse Y, Maltais F, Road J, Rocker G, Sin D, Sinuff T, Voduc N: **Canadian Thoracic Society recommendation for management of chronic obstructive pulmonary disease - 2007 update.***Can Respir J* 2007, **14:**5b–32b.

22. Nava S, Fanfulla F: *Ventilazione meccanica non invasiva. Come, quando e perché.* Milano: Springer-Verlag Italia; 2010.

References regarding paragraph "Integrated hospital-community management of patients with severe COPD"

1. Statement ATS: **Standards for the diagnosis and care of patients with chronic obstructive pulmonary disease.***Am J Respir Crit Care Med* 1995, **5:**S77–S120.

2. ATS ERS: **statement.***Eur Respir J* 2004, **23:**932–946.

3. Corrado A, Roussus C, Ambrosino N, Confalonieri M, Cuvelier A, Elliott M, Ferrer M, Gorini M, Gurkan O, Muir JF, Quareni L, Robert D, Rodenstein D, Rossi A, Schoenhofer B, Simonds AK, Strom K, Torres A, Zakynthinos S; European Respiratory Society Task Force on epidemiology of respiratory intermediate care in Europe: **Respiratory intermediate care units: an European survey.***Eur Respir J* 2002, **20:**1343–1350.

4. Corrado A, Ambrosino N, Cavalli A, Sturani C: **Unità di terapia intensiva respiratoria: update.***RassPatAppRespir* 2004, **19:**18–34.

5. *Linee Guida "Insufficienza Respiratoria" Regione Toscana.*2010.

6. Statement on Home Care for Patients with Respiratory Disorders: **This official statement of the American Thoracic Society was approved by the ATS board of Directors December 2005.***Am J RespirCrit Care Med* 2005, **171:**1443–1464.

7. Farrero E, Escarrabill J, Prats E, Maderal M, Manresa E: **Impact of a hospital-based home-care program on the management of COPD patients receiving long-term oxygen therapy.***Chest* 2001, **119:**364–369.

8. Hermiz O, Comino E, Marks G, Daffurn K, Wilson S, Harris M: **Randomised controlled trial of home based care of patients with chronic obstructive pulmonary disease.***BMJ* 2002, **325:**938–940.

9. Lanken PN, Terry PB, Delisser HM, Fahy BF, Hansen-Flaschen J, Heffner JE, Levy M, Mularski RA, Osborne ML, Prendergast TJ, Rocker G, Sibbald WJ, Wilfond B, Yankaskas JR; ATS End-of-Life Care Task Force: **An official American Thoracic Society clinical policy statement: palliative care for patients with respiratory diseases and critical illnesses.***Am J Respir Crit Care Med* 2008, **177:**912–927.

10. Curtis JR: **Palliative and end of life care for patients with severe COPD.***EurRespir J* 2008, **32:**796–803.

11. **Cure palliative dei pazienti con patologie respiratorie croniche avanzate non oncologiche.** 2011. Position Paper AIPO-SIAARTI-ARIR.

12. *D.M. del MURST 20/4/90.*

13. *Piano Sanitario Nazionale 2011–2013.* www.salute.gov.it

General bibliography regarding paragraph "Integrated hospital-community management of patients with severe COPD"

Jaana M, Paré G, Sicotte C: **Home telemonitoring for respiratory conditions: a systematic review.***Am J Manag Care* 2009, **15:**313–320.

*Linee Guida "Insufficienza Respiratoria" Regione Toscana.* 2010.

Statement ATS: **Standards for the diagnosis and care of patients with chronic obstructive pulmonary disease.***Am J Respir Crit Care Med* 1995, **5:**S77–S121.

Statement on Home Care for Patients with Respiratory Disorders: **This official statement of the American Thoracic Society was approved by the ATS board of Directors December 2005.***Am J Respir Crit Care Med* 2005, **171:**1443–1464.

Corrado A, Roussus C, Ambrosino N, Confalonieri M, Cuvelier A, Elliott M, Ferrer M, Gorini M, Gurkan O, Muir JF, Quareni L, Robert D, Rodenstein D, Rossi A, Schoenhofer B, Simonds AK, Strom K, Torres A, Zakynthinos S; European Respiratory Society Task Force on epidemiology of respiratory intermediate care in Europe: **Respiratory intermediate care units: an European survey.***EurRespir J* 2002, **20:**1343–1350.

Farrero E, Escarrabill J, Prats E, Maderal M, Manresa F: **Impact of a hospital-based home-care program on the management of COPD patients receiving long-term oxygen therapy.***Chest* 2001, **119:**364–369.

Hermiz O, Comino E, Marks G, Daffurn K, Wilson S, Harris M: **Randomised controlled trial of homebased care of patients with chronic obstructive pulmonary disease.***BMJ* 2002, **325:**938–940.

Jaana M, Paré G, Sicotte C: **Home telemonitoring for respiratory conditions: a systematic review.***Am J Manag Care* 2009, **15:**313–320.

Dal Negro RW, Goldberg AI (Eds): *Home Long-Term Oxygen Treatment in Italy. The Additional Value of Telemedicine".* Berlin Heidelberg. Springer Publ; 2005:71–85.

Vitacca M, Mazzù M, Scalvini S: **Socio-technical and organizational challenges to wider e-Health implementation.***ChronRespir Dis* 2009, **6:**91.

Vitacca M, Bianchi L, Guerra A, Fracchia C, Spanevello A, Balbi B, Scalvini S: **Tele-assistance in chronic respiratory failure patients: a randomised clinical trial.***Eur Respir J* 2009, **33:**411–418.

Vitacca M, Comini L, Tentorio M,Assoni G, Trainini D, Fiorenza D, Morini R, Bruletti G, Scalvini S:**A pilot trial of telemedicine-assisted, integrated care for patients with advanced amyotrophic lateralsclerosis and their caregivers.***J TelemedTelecare* 2010, **16:**83–88.

Piano Sanitario Nazionale 2011–2013.

D.M. del MURST 20/4/90.

References regarding paragraph "Palliative and end of life care in COPD"

Lanken PN, Terry PB, Delisser HM, Fahy BF, Hansen-Flaschen J, Heffner JE, Levy M, Mularski RA, Osborne ML, Prendergast TJ, Rocker G, Sibbald WJ, Wilfond B, Yankaskas JR; ATS End-of-Life Care Task Force: **An official American Thoracic Society clinical policy statement: palliative care for patients with respiratory diseases and critical illnesses.***Am J Respir Crit Care Med* 2008, **177:**912–927.

Curtis JR: **Palliative and end of life care for patients with severe COPD.***Eur Respir J* 2008, **32:**796–803.

**Cure palliative dei pazienti con patologie respiratorie croniche avanzate non oncologiche.** 2011. Position Paper AIPO-SIAARTI-ARIR.

GOLD Global Initiative for Chronic Obstructive Lung Disease: *Global Strategy for the Diagnosis, Management, and Prevention of Chronic Obstructive Pulmonary Disease*. Updated 2011.

Centre NCG: *Chronic Obstructive Pulmonary Disease: Management of Chronic Obstructive Pulmonary Disease in Adults in Primary and Secondary Care.* London: National Clinical Guideline Centre; 2010. Available from: http://guidance.nice.org.uk/CG101/Guidance/pdf/English.

Celli B, MacNee W, and committee members: **Standards for the diagnosis and treatment of patients with COPD: a summary of the ATS/ERS position paper.***Eur Respir J* 2004, **23:**932–946.

Brusasco V, Crapo R, Viegi G; American Thoracic Society; European Respiratory Society: **Coming together: the ATS/ERS consensus on clinical pulmonary function testing.***Eur Respir J* 2005, **26:**1–2. 153–161; 319–338; 511–522; 720–735; 948–968.

**Screening for chronic obstructive pulmonary disease using spirometry: U.S. preventive services task force recommendation statement.***Ann Intern Med* 2008, **148:**529–534.

Qaseem A, Wilt TJ, Weiberger SE, Hanania NA, Criner G, van der Molen T, Marciniuk DD, Denberg T, Schünemann H, Wedzicha W, MacDonald R, Shekelle P; American College of Physicians; American College of Chest Physicians; American Thoracic Society; European Respiratory Society: **Diagnosis and management of stable chronic obstructive pulmonary disease: a clinical practice guideline from the ACP, ACCP, ATS and ERS.***Ann Intern Med* 2011, **155:**179–191.

## Competing interests

The authors declare that they have no competing interests.

